# Reply to the letter: will non-invasive methods replace bone biopsy in patients with chronic kidney disease?

**DOI:** 10.1590/2175-8239-JBN-2025-0385rpen

**Published:** 2026-07-31

**Authors:** Mariana Fernandes Diz Lopes, Bernardo José Cardoso Fernandes, Maria Teresa Almeida e Sousa Martins da Rocha, Ricardo de Morais Pereira

**Affiliations:** 1 Unidade Local de Saúde de São João, Serviço de Reumatologia, Porto, Portugal.; 2 Universidade do Porto, Faculdade de Medicina, Departamento de Medicina, RISE-Health, Porto, Portugal.; 3 Unidade Local de Saúde de São João, Serviço de Nefrologia, Porto, Portugal.

We thank Araujo et al.^1^ for their thoughtful comments on our recently published study that evaluated the relationship between bone histomorphometry, circulating bone biomarkers, and FRAX^®^ in predicting fractures in patients with predialysis chronic kidney disease (CKD)^2^.

We fully agree with the authors that bone biopsy remains the gold standard for the evaluation of renal osteodystrophy and continues to play a crucial role in the diagnosis of complex bone disorders in CKD. As highlighted in their letter, histomorphometric analysis is uniquely capable of identifying conditions such as aluminum-related bone disease, which may have important therapeutic implications and cannot be detected by non-invasive methods.

However, our study was not designed to challenge the clinical value of bone biopsy. Rather, our aim was to explore whether histomorphometric subtypes of renal osteodystrophy were associated with fracture occurrence in a cohort of patients with predialysis CKD. In our population, we did not observe a significant association between histomorphometric subtypes and fracture incidence, although we did observe a trend toward lower bone volume in patients who developed fractures during follow-up.

Importantly, the absence of an association between histological subtype and fracture risk should not be interpreted as evidence that bone biopsy lacks clinical relevance. This procedure provides comprehensive information on bone turnover, mineralization, and bone volume that cannot be obtained from the clinical history, imaging studies, or circulating biomarkers. We also noted in our study that the small number of patients limits the evaluation of fractures and fracture risk predictors.

We also acknowledge that epide­miological differences may influence the clinical applicability of these findings. As emphasized by Araujo et al.^1^, the prevalence of aluminum-related bone disease may still be significant in some regions of Brazil, reinforcing the continued relevance of bone biopsy in this specific clinical context. Furthermore, the method remains valuable, even in a European context, particularly when treatment decisions depend on accurately distinguishing between high- and low-turnover bone disease and when there is a possibility of a mineralization defect^3,4^.

At the same time, we believe that, in some selected clinical scenarios, bone biopsy should not necessarily be considered a prerequisite for initiating treatment aimed at reducing fracture risk. In this context, our results highlight the potential utility of FRAX^®^ as a pragmatic and accessible tool for fracture risk stratification in patients with CKD, particularly when bone mineral density or bone biopsy data are not available.

Based on current evidence, including our own data, bone biopsy, clinical risk assessment tools such as FRAX^®^, and circulating bone biomarkers should be viewed as complementary approaches in the evaluation of skeletal health in CKD patients.

## Data Availability

The data that support the findings of this study are available from the corresponding author upon reasonable request.
