# Supplementary material for: Reply to the letter: will non-invasive methods replace bone biopsy in patients with chronic kidney disease?
Source: J Bras Nefrol. 2026 Jul 31;48(3):e20250385rp. doi: 10.1590/2175-8239-JBN-2025-0385rpen (PMC13440858; doi:10.1590/2175-8239-JBN-2025-0385rpen)
Supplement: Translation

# Translation: Resposta à carta: os métodos não invasivos substituirão a biópsia óssea nos pacientes com doença renal crônica?

**DOI:** 10.1590/2175-8239-JBN-2025-0385rppt

**Authors:** Mariana Fernandes Diz Lopes, Bernardo José Cardoso Fernandes, Maria Teresa Almeida e Sousa Martins da Rocha, Ricardo de Morais Pereira

**Affiliations:** 1 Unidade Local de Saúde de São João, Serviço de Reumatologia, Porto, Portugal.; 2 Universidade do Porto, Faculdade de Medicina, Departamento de Medicina, RISE-Health, Porto, Portugal.; 3 Unidade Local de Saúde de São João, Serviço de Nefrologia, Porto, Portugal.

Agradecemos a Araujo et al.^1^ pelos comentários pertinentes sobre o nosso estudo recentemente publicado, que avaliou a relação entre histomorfometria óssea, biomarcadores ósseos e FRAX^®^ na predição de fraturas em pacientes com doença renal crônica (DRC) em fase pré-diálise^2^.

Concordamos plenamente com os autores que a biópsia óssea continua sendo o padrão-ouro para a avaliação da osteodistrofia renal e mantém um papel crucial no diagnóstico de doenças ósseas complexas na DRC. Tal como destacado em sua carta, a análise histomorfométrica é o único método capaz de identificar condições como a doença óssea relacionada ao alumínio, que pode ter implicações terapêuticas importantes e não pode ser detectada por métodos não invasivos.

Contudo, nosso estudo não foi concebido para questionar o valor clínico desse exame. Nosso objetivo foi explorar se os subtipos histomorfométricos de osteodistrofia renal estavam associados à ocorrência de fraturas em uma coorte de pacientes com DRC em pré-diálise. Em nossa população, não observamos uma associação significativa entre os subtipos histomorfométricos e a incidência de fraturas, embora tenha sido observada tendência a menor volume ósseo nos pacientes que desenvolveram fraturas durante o seguimento.

É importante destacar que a ausência de associação entre o subtipo histológico e o risco de fratura não deve ser interpretada como evidência de que a biópsia óssea careça de relevância clínica. A biópsia óssea fornece informações abrangentes sobre o *turnover* ósseo, a mineralização e o volume ósseo, que não podem ser obtidas por meio da história clínica, de exames de imagem ou de biomarcadores circulantes. Também foi referido em nosso estudo que o reduzido número de pacientes limita a avaliação de fraturas e de preditores de risco de fratura.

Reconhecemos também que as diferenças epidemiológicas podem influenciar a aplicabilidade clínica desses resultados. Como enfatizado por Araujo et al.^1^, a prevalência de doença óssea relacionada ao alumínio pode ainda ser significativa em algumas regiões do Brasil, reforçando a relevância contínua da biópsia óssea nesse contexto clínico específico. Além disso, tal procedimento continua sendo importante, mesmo no contexto europeu, particularmente quando as decisões terapêuticas dependem da distinção precisa entre doença óssea de alto e baixo *turnover* e quando existe a possibilidade de defeitos de mineralização^3,4^.

Ao mesmo tempo, consideramos que, em alguns cenários clínicos selecionados, o método não deve ser necessariamente considerado um pré-requisito para iniciar o tratamento direcionado à redução do risco de fratura. Nesse contexto, nossos resultados destacam a potencial utilidade do FRAX^®^ como uma ferramenta pragmática e acessível para a estratificação do risco de fratura em pacientes com DRC, particularmente quando não estão disponíveis dados de densidade mineral óssea ou de biópsia óssea.

Com base nas evidências atuais, incluindo nossos próprios dados, a biópsia óssea, as ferramentas de avaliação de risco clínico, como o FRAX^®^, e os biomarcadores ósseos devem ser considerados abordagens complementares na avaliação da saúde esquelética em pacientes com DRC.

## Data Availability

Os dados que dão suporte aos resultados deste estudo estão disponíveis com os autores correspondentes, mediante solicitação razoável.
